# Strategies to Overcome Resistance to Osimertinib in EGFR-Mutated Lung Cancer

**DOI:** 10.3390/ijms26072957

**Published:** 2025-03-25

**Authors:** Donatella Romaniello, Alessandra Morselli, Ilaria Marrocco

**Affiliations:** 1 Department of Medical and Surgical Sciences (DIMEC), University of Bologna, Via Massarenti 9, 40138 Bologna, Italy; donatella.romaniello@unibo.it (D.R.); alessandra.morselli4@unibo.it (A.M.); 2 IRCCS Azienda Ospedaliero-Universitaria di Bologna, Via Massarenti 9, 40138 Bologna, Italy; 3 Department of Life Sciences and Public Health, Università Cattolica del Sacro Cuore, 00168 Rome, Italy

**Keywords:** NSCLC, EGFR, osimertinib, drug resistance, bispecific antibodies, combination therapy

## Abstract

Non-small-cell lung cancer (NSCLC) represents the most common type of lung cancer. The majority of patients with lung cancer characterized by activating mutations in the epidermal growth factor receptor (EGFR), benefit from therapies entailing tyrosine kinase inhibitors (TKIs). In this regard, osimertinib, a third-generation EGFR TKI, has greatly improved the outcome for patients with EGFR-mutated lung cancer. The AURA and FLAURA trials displayed the superiority of the third-generation TKI in both first- and second-line settings, making it the drug of choice for treating patients with EGFR-mutated lung cancer. Unfortunately, the onset of resistance is almost inevitable. On-target mechanisms of resistance include new mutations (e.g., C797S) in the kinase domain of EGFR, while among the off-target mechanisms, amplification of MET or HER2, mutations in downstream signaling molecules, oncogenic fusions, and phenotypic changes (e.g., EMT) have been described. This review focuses on the strategies that are currently being investigated, in preclinical and clinical settings, to overcome resistance to osimertinib, including the use of fourth-generation TKIs, PROTACs, bispecific antibodies, and ADCs, as monotherapy and as part of combination therapies.

## 1. Lung Cancer

The latest cancer statistics, released by the International Agency for Research on Cancer (IARC) for the year 2022, identified lung cancer as the most frequently diagnosed type of cancer (12.4% of total cases), with 2.5 million new cases, and as the leading cause of cancer-related-death worldwide (18.7% of total cancer death) [1,2]. The five-year survival rate remains below 20% due to late-stage diagnoses. However, the European Commission and several other countries are now planning to implement several screening programs in hopes of improving the outcome for lung cancer patients [1].

Histologically, lung cancer is a heterogeneous disease classified into two major categories: small-cell lung cancer (SCLC) and non-small-cell lung cancer (NSCLC), which accounts for 85% of all diagnosed cases [3]. In NSCLC, adenocarcinoma is the most common type (about 75%), while the incidence of squamous cell carcinoma has reduced to 18% of cases partially because of prevention measures, such as anti-smoke campaigns adopted in high-income countries [3,4]. However, this proportion may vary depending on gender and geography, and in some countries, such as Spain and the Netherlands, squamous cell carcinoma is, on the contrary, more frequent compared to adenocarcinoma [5,6]. The epidermal growth factor receptor (EGFR), among the first driver oncogenes identified in NSCLC, appears to be commonly altered in non-smoker patients with lung adenocarcinoma and represents a predictive biomarker for targeted therapy with EGFR-specific tyrosine kinase inhibitors (TKIs) [7,8]. In the context of NSCLC, several other targetable driver oncogenes have been identified, including serine/threonine-protein kinase b-raf (BRAF; 3–8%), Kirsten rat sarcoma viral oncogene homolog (KRAS; 20–30%), hepatocyte growth factor receptor (MET; 3%), human epidermal growth factor receptor 2 (HER2; 2%), anaplastic lymphoma kinase (ALK; 1–7%), ROS1 proto-oncogene receptor tyrosine kinase (ROS1; 1–2%), tropomyosin receptor kinase (NTRK; <1%), rearranged during transfection (RET; 1–2%), and neuregulin 1 (NRG1) fusion variants (0,14%) [9,10,11,12,13,14,15]. Meanwhile, neurofibromin 1 (NF1), a tumor suppressor gene that negatively regulates Ras signaling, has been found mutated in 10% of NSCLC cases and appears to be a predictive biomarker of response to immune checkpoint inhibitors [16,17,18].

## 2. EGFR-Mutated NSCLC: The Rise of TKI Treatments

EGFR (also known as HER1, ErbB1) is considered one of the most common driver oncogenes in various solid tumors including NSCLC, where it is found to be altered in approximately 29% of patients, ranging from 12% in Western countries to 47% in Asia–Pacific regions [19,20,21]. Ubiquitously expressed in several tissue types, EGFR plays a key role in embryogenesis and post-natal development [22]. It belongs to the ErbB/HER family of receptor tyrosine kinases (RTKs), which also includes HER2 (ErbB2), HER3 (ErbB3), and HER4 (ErbB4) [23]. Several ligands bind to the HER family: epidermal growth factor (EGF), transforming growth factor-alpha (TGF-α), epigen (EPGN), and amphiregulin (AR) are exclusively EGFR ligands, while betacellulin (BTC), heparin-binding epidermal growth factor (HB-EGF), and epiregulin (EPR) are shared with HER4. Additionally, neuregulins (NRGs) 1–4 bind to HER4, while only NRG1–2 serve as ligands for HER3 [24]. Canonically, signaling activation starts upon ligand binding, and the consequent conformational changes prompt the monomeric “activator kinase” to dimerize, forming either homo- or heterodimers with other HER family members. This interaction enables the “receiver kinase” to trans-phosphorylate specific tyrosine residues on the activator’s C-lobe, initiating a cascade of downstream signaling events, which ultimately leads to the activation of key intracellular pathways, including the Ras/Raf/mitogen-activated protein kinase (MAPK) pathway, the phosphatidylinositol 3-kinase (PI3K)/AKT/mammalian target of rapamycin (mTOR) pathway, and the signal transducer and activator of transcription (STAT) pathway [25]. While all ErbB receptors share common features, there are important differences: HER2 is often referred to as an “orphan receptor” because it lacks a direct ligand, whereas HER3 has low or null kinase activity, which explains the strong cooperation between all family members.

In NSCLC, EGFR genetic alterations are mostly represented by several deletions in exon 19 (Del19) and by the point mutation L858R in exon 21. Together, these mutations account for approximately 85% of all EGFR mutations in lung cancer, and for this reason, they are often referred to as common or canonical mutations. The most frequent exon 19 deletions are represented by p.E746_A750del (64.6%), followed by p.L747_P753 > S (8.4%), p.L747_T751del (4.0%), p.L747_A750 > P (3.4%), p.E746-S752 > V, (3.2%), p.E746_S752 > V (1.6%), and p.L747_S752del (1.4%) [26]. The remaining 15% are represented by rare or uncommon mutations, including point mutations (e.g., L861Q in exon 21, G719X in exon 18, S768I in exon 20, and L861Q in exon 21), insertions in exon 20 (Ins20), deletions in exon 18 (Del 18), and other rare alterations like mutations in exon 2–15, gene fusion, and EGFR-kinase domain duplication [27,28,29,30,31,32,33]. The presence of such EGFR alterations, which are usually mutually exclusive with mutations in other oncogenes, such as KRAS, induces constitutive, ligand-independent activation of the receptor and its downstream signaling pathways, promoting tumor cell survival and proliferation. Most importantly, the presence of canonical mutations indicates specific tumor cell sensitivity to EGFR TKIs, providing treatment benefits for patients with metastatic NSCLC (mNSCLC). A list of drugs utilized in clinics for EGFR-mutated lung cancer is presented in Table 1.

The first-generation EGFR TKI gefitinib was approved in 2015 by the FDA as first-line therapy for NSCLC patients harboring common EGFR activating mutations (Del19 or L858R). The superiority of gefitinib, compared to chemotherapy, was demonstrated in several phase 3 clinical trials (Table 1) [34,35,36,37,38,39,59]. Together with gefitinib, other first-generation TKIs were developed and approved, including erlotinib and icotinib (China only). This class of drugs comprises reversible inhibitors that, by competitively binding to the ATP binding pocket of EGFR, block receptor autophosphorylation, and, consequently, activation of downstream survival pathways [60,61]. Soon after, to overcome the limits due to their reversible nature and, primarily, the acquisition of the T790M gatekeeper mutation in exon 20, which causes resistance in 50–60% of patients within 9–15 months of treatment, second-generation irreversible TKIs, comprising afatinib, dacomitinib, and neratinib, were developed [44,50,62,63]. However, clinical efficacy of these inhibitors in T790M mutant patients was not achieved, most likely because the presence of the gatekeeper mutation slows down the formation of the covalent bond of irreversible inhibitors, thus limiting their cytotoxic activities [64]. Moreover, the fact that second-generation TKIs fail to spare wild-type EGFR makes it impossible to use the effective dose without causing significant adverse effects. For these reasons, third-generation inhibitors have been formulated to selectively target T790M-EGFR. These pyrimidine derivatives are able to covalently bind the cysteine residue 797, while sparing wild-type EGFR. In November 2015, based on the data coming from the AURA3 phase 3 trial, osimertinib (also known as AZD9291) was approved by the FDA (Food and Drug Administration) as second-line treatment for locally advanced or metastatic EGFR-T790M-positive NSCLC patients who progressed after first-generation TKIs [46,47,65,66]. Thereafter, the phase 3 FLAURA trial demonstrated the superiority of osimertinib, when compared to first-generation TKIs, for patients with NSCLC expressing one of the two common EGFR mutations (Del19 or L858R). The trial showed improved progression-free survival (PFS) and overall survival (OS), reducing the risk of death by 20% and reducing side effect frequency [48,49]. Moreover, it was shown that osimertinib can reduce the risk of central nervous system metastasis progression by 52%, due to its ability to cross the blood–brain barrier [49,67]. In summary, osimertinib demonstrated the ability to irreversibly inhibit EGFR harboring Del19 and L858R mutations while sparing wild-type EGFR, resulting in fewer side effects and long-term treatment; it is able to effectively overcome T790M-mediated resistance, and to cross the blood–brain barrier, providing benefits in patients suffering from brain metastases. Thus, given the outstanding results from the FLAURA trial and the numerous advantages compared to other generations of EGFR TKIs, osimertinib is currently recommended in front-line treatment for locally advanced unresectable EGFR-mutated (Del19 and L858R) NSCLC [68,69]. Recent advancements, based on the primary findings of the FLAURA2 and MARIPOSA studies, recommend combination therapies in first-line settings. Both osimertinib combined with platinum-based chemotherapy and lazertinib, a third-generation EGFR TKI, with amivantamab, a bispecific antibody co-targeting EGFR and MET, have demonstrated superiority compared to osimertinib alone [70,71].

Of note, despite the fact that EGFR TKIs are utilized for the treatment of lung cancer with EGFR canonical mutations, there may be differences, in terms of drug response, among various EGFR mutants. In this respect, Marrocco et al. observed that the L858R mutation was more sensitive to a monoclonal antibody targeting EGFR, namely cetuximab, compared to deletions in exon 19 [72]. These differences in drug response could be related to the mechanisms of kinase activation: while the Del19-EGFR mutant is active as a monomer, L858R-EGFR requires dimerization [73]. Similarly, different exon 19 deletion variants may exhibit distinct behaviors. For example, p.L747_A750 > P deletion has been associated with poorer PFS compared to p.E746_A750del when treated with osimertinib [74]. Moreover, a recent study, investigating the mutational landscape in approximately 16,700 patients with EGFR-mutant NSCLC, stratified EGFR mutations in four subgroups based on the structure of the mutant and the sensitivity to kinase inhibitors. This structure–function approach could help predict responsiveness to TKIs [75].

Despite the excellent clinical response to osimertinib, resistance inevitably occurs. Since its first approval, in 2015, multiple EGFR-dependent and -independent mechanisms of resistance have been identified. However, due to high tumor heterogeneity among patients, strategies to prevent and/or overcome resistance to this third-generation EGFR TKI remain an active area of research.

## 3. Acquired Resistance to Osimertinib

**Figure 1:**
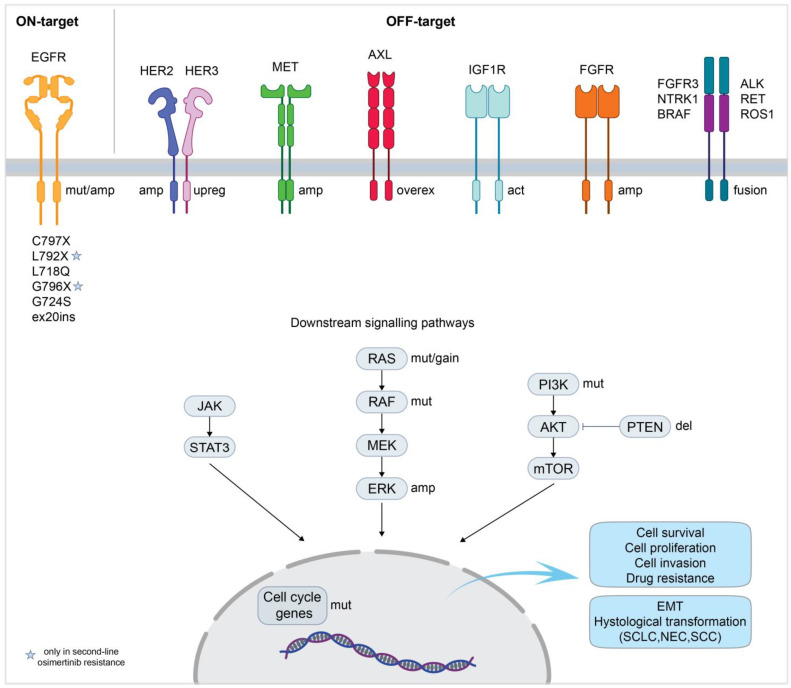
Mechanisms of resistance to osimertinib in NSCLC. The cartoon illustrates the common mechanisms of resistance in patients treated with osimertinib, both in first- and second-line therapy. Resistance mechanisms can be subdivided into two categories: “on-target”, referring to alteration in EGFR, and “off-target”, involving alternative signaling pathways [77,78,79,80,81]. Mut, mutation; amp, amplification; upreg, upregulation; overex, overexpression; act, activation; del, deletion; gain, gain of copy number; EMT, epithelial-to-mesenchymal transition; SCLC, small-cell lung cancer; NEC, neuroendocrine carcinoma; SCC, squamous cell carcinoma.

The advantages of using osimertinib in the treatment of EGFR-mutated NSCLC have been largely demonstrated by the AURA3 and FLAURA studies, in both second- and first-line settings, respectively [47,48,49]. Despite great efficacy, most patients eventually develop drug resistance within 10–20 months [76]. Drug resistance in patients who received osimertinib in second-line settings, after failure of first-generation inhibitors, can be attributable to on- or off-target mechanisms (Figure 1).

EGFR mutations are responsible for resistance to second-line osimertinib in 22% of patients. The majority of acquired mutations involve the cysteine residue 797, with the C797S being the most representative one (14%) [81]. Because osimertinib requires the formation of a covalent bond with the cysteine 797 in the ATP binding pocket of EGFR [82], the substitution of this residue impairs its binding to the receptor. Other mutated residues detected in patients resistant to osimertinib are listed in Figure 1 and include C796S, L718Q, C797G, and L792X [81,83,84]. Interestingly, it has been shown that resistance mutations can preferentially occur with one of the two most common activating mutations. For instance, while C797S is more frequent in Del19+ tumors [85], L718V/Q and G724S selectively co-occur with L858R- and Del19-EGFR, respectively [86,87]. Moreover, EGFR gene amplification has been described as a possible mechanism of resistance to osimertinib [88,89]. Among off-target mechanisms of resistance, the most common is represented by the amplification of MET (18% of patients), followed by the amplification of HER2 and alterations in downstream signaling molecules (MAPK and PI3K pathways) [81]. Additionally, histological transformations from non-small-cell to small-cell lung cancer (SCLC), large-cell neuroendocrine carcinoma (NEC), or squamous cell carcinoma have been reported as potential mechanisms of resistance to second-line osimertinib in case reports [90,91,92,93,94]. Resistance to first-line osimertinib shares similarities with second-line treatment (Figure 1). While EGFR-T790M mutation was not detected, the most common reported mechanisms of resistance include MET amplification (16%) and the C797S mutation (6%) [80]. HER2 amplification and alterations in the MAPK and PI3K pathways have also been described following first-line osimertinib [80,95]. Oncogenic fusions affecting tyrosine kinases, such as RET and ALK, have also been identified in patients resistant to osimertinib [80,81,96]. Additionally, alterations in cell cycle regulators (e.g., cyclin D amplification, CDK4/6 amplification) have also been reported in patients who progressed to osimertinib in both first- and second-line settings [80,81]. Additional mechanisms involved in resistance to osimertinib are illustrated in Figure 1 and will be discussed in the following sections.

It has been demonstrated that molecular profiling of osimertinib resistance using both tumor tissue and circulating tumor DNA (ctDNA) helps identify patients who derive the greatest benefit from subsequent targeted treatment combinations [97]. Accordingly, the ESMO recommendation for patients with EGFR-mutated NSCLC who progress on TKI therapy is to assess actionable mechanisms of resistance on tissue rebiopsy when possible or on circulating tumor DNA (ctDNA) when no tissue is available [98]. Additionally, a relevant factor to consider when selecting further therapies after osimertinib failure is the co-occurrence of multiple mechanisms of resistance, a common feature observed in patients after first- or second-line osimertinib [80,81].

## 4. Strategies to Overcome On-Target Resistance to Osimertinib (See Table 2)

### 4.1. Fourth-Generation EGFR TKIs

As an effort to overcome C797S-mediated resistance, which has been observed in a large fraction of patients who progressed after first- or second-line osimertinib (6 and 14%, respectively), fourth-generation EGFR tyrosine kinase inhibitors are under development. This new category of drugs includes both ATP-competitive inhibitors, acting with a mechanism similar to previous generation drugs, and allosteric inhibitors, which have the potential advantage of reducing the risk of on-target resistance, a common issue with competitive inhibitors.

Among the first allosteric inhibitors developed to overcome C797S-mediated resistance, EAI001 and EAI045 were proven to be able to bind and block EGFR in an inactive conformation. However, EAI045 was able to inhibit the proliferation of cell lines and tumor growth of models expressing L858R/T790M- and L858R/T790M/C797S-EGFR, only when combined with dimerization blocking antibodies, like cetuximab [99]. A new allosteric inhibitor, namely JBJ-04-125-02, was developed to overcome this problem. Indeed, single agent JBJ-04-125-02 was active in vitro and in vivo against models of lung cancer carrying L858R/T790M- and L858R/T790M/C797S-EGFR [100]. Of note, JBJ-04-125-02 showed synergy with osimertinib, thus suggesting a potential benefit of combination therapies entailing an allosteric inhibitor and an ATP-competitive inhibitor for EGFR-mutated lung cancer [100,101]. An important factor to consider is that neither EAI045 nor JBJ-125–02 was able to inhibit Exon19Del-EGFR, due to the inaccessibility of the allosteric pocket in this mutant [99,100]. An example of a fourth-generation inhibitor able to target not only L858R- but also Del19-expressing tumors is represented by BLU-945. Importantly, this potent, reversible, inhibitor is active against EGFR carrying resistance mutations (T790M ± C797S), while sparing the wild-type receptor [102]. Preclinical studies showed that this potent reversible EGFR inhibitor was able to block the growth of cell line xenografts and patient-derived xenografts (PDXs), expressing mutant EGFR, as a single agent, as well as combined with osimertinib [103].

The number of fourth-generation inhibitors is increasing, and several have entered clinical evaluation (Table 2). BLU-945 entered a phase 1/2 clinical trial (SYMPHONY), which showed tumor shrinkage in patients associated with a safety profile [103,104]. However, its development was stopped in January 2024 [105]. A similar fate was encountered by BLU-701. This inhibitor showed in vivo efficacy against models of lung cancer expressing Exon19Del-, L858R-, and C797S-EGFR [106,107], but the phase I/II clinical trial (HARMONY) was terminated due to a lack of efficacy. BDTX-1535 is an irreversible, blood–brain-barrier-permeable EGFR inhibitor able to target C797S-EGFR that is currently under clinical evaluation in a phase 1/2 dose expansion trial (NCT05256290). Preliminary data on 19 C797S-positive patients showed a response rate of 42% [108,109]. Other fourth-generation inhibitors under clinical evaluation are listed in Table 2.

### 4.2. Combining Osimertinib with Other EGFR TKIs

One possible way to avoid the occurrence of resistance is represented by combinatorial approaches. In this regard, knowing the mechanism of resistance to osimertinib is crucial for selecting the appropriate treatment.

To fight on-target resistance, the combination of osimertinib with EGFR allosteric inhibitors might be beneficial, considering that they target different sites of the receptor. For instance, the combination of osimertinib and the fourth-generation TKI described above, BLU-945, showed efficacy in both in vitro and in vivo models of C797S-positive EGFR-mutated lung cancer, as well as in patients from the SYMPHONY trial [103,104].

In the context of resistance to second-line osimertinib, patients might simultaneously express the T790M and C797S mutations in EGFR. In this case, the group of Engelman demonstrated that if the two mutations occur on opposite alleles (in trans), the receptor can be inhibited by a combination of first- and third-generation EGFR TKIs, whereas if the two mutations are in cis, the combination is not effective [110]. Moreover, a transient clinical response to the combination of first- and second-generation TKIs in patients expressing in-trans T790M/C797S was observed in two case reports [111,112].

Importantly, C797S-mediated resistance to first-line osimertinib could be overcome by using previous-generation EGFR TKIs [110]. The efficacy of using erlotinib following C797S-mediated resistance to first-line osimertinib was demonstrated in preclinical in vitro models, and a transient response was observed in a case report [113]. Additionally, preliminary data from a phase 1/2 clinical trial in patients who received first-line osimertinib plus gefitinib show an objective response rate (ORR) of 85.2% [114]. The same combination is under evaluation in the ORCHARD study, in patients who progressed after first-line osimertinib via mutations affecting cysteine 797 [115].

### 4.3. PROTACs

Another possible approach to counteract on-target resistance to osimertinib could involve receptor degradation. In this regard, a new technology, namely proteolysis targeting chimera (PROTAC), could represent a valid option to fight drug resistance. PROTACs are bifunctional molecules that simultaneously bind to a target protein and to an E3 ubiquitin ligase. The result is the ubiquitination of the target protein, which typically is represented by an oncogene, followed by proteasomal degradation, with a consequent decrease in protein levels [116,117]. Since the first small-molecule-based PROTAC, targeting the androgen receptor (AR), was reported in 2008 by the Crews group [118], several more, targeting a variety of oncogenic proteins (e.g., cyclin-dependent kinases, BET proteins, estrogen receptor (ER), the serine–threonine kinase RIPK2, and focal adhesion kinase (FAK)), were developed [119,120,121,122,123,124], and some have entered clinical trials [125]. For instance, the ER degrader vepdegestrant showed clinical activity and a safety profile in a phase 1/2 study, and it is currently under clinical evaluation in the VERITAC-2 phase 3 trial in pretreated patients with ER+/HER2- advanced breast cancer [126].

The first PROTACs targeting EGFR were developed by the group of Crews. They showed that a PROTAC utilizing gefitinib as a protein binder was able to reduce EGFR protein levels in NSCLC cells expressing one of the two EGFR-activating mutations (Del19 or L858R) [127]. The gefitinib-based degrader, on the other hand, did not affect the wild-type receptor, thus indicating that mutant EGFR, a known oncogene, could selectively be targeted, avoiding potential side effects due to inhibition of the wild-type receptor. The same group demonstrated the ability of an afatinib-based degrader to reduce the levels of the receptor in a cell line carrying L858R/T790M-EGFR [127]. A PROTAC based on the structure of another second-generation EGFR TKI, namely dacomitinib, was developed by the group of Zhu. This degrader was able to decrease EGFR protein levels and block the activation of downstream signaling pathways in cells expressing Del19-EGFR, while it was not active against the wild-type receptor or other EGFR mutants. This compound was also able to reduce the growth of a Del19-EGFR expressing cell line, in vitro and in vivo [128]. Third-generation TKI-based degraders were also developed. Zhang’s group reported an osimertinib-derived PROTAC able to reduce EGFR protein levels and inhibit the growth of Del19- and L858R/T790M-EGFR-expressing cell lines [129]. In order to counteract C797S-mediated resistance to osimertinib, degraders based on the structure of allosteric EGFR inhibitors have also been developed. Gray’s laboratory reported the efficacy of an allosteric EGFR degrader in inhibiting the growth of cell lines expressing C797S- or L718Q-EGFR, two mutations that are observed in patients who progressed to osimertinib [130]. A list of EGFR PROTACs based on the structure of first-, second-, third-, and fourth-generation EGFR TKIs has been reviewed in [131]. Additionally, because it has been shown that the ALK inhibitor brigatinib is active against C797S-EGFR expressing models in vitro and in vivo [132], brigatinib-based degraders have also been developed. This type of PROTAC showed time- and dose-dependent degradation of C797S-EGFR and anti-proliferative activity in cell lines expressing this mutant form of the receptor, as well as in C797S-positive cell-derived and patient-derived xenografts [133,134].

Considering that the binding ability of small TKIs depends on the mutational state of the receptor, an alternative approach when designing EGFR degraders could be the employment of antibody-based PROTACs. In this respect, a cetuximab-based EGFR degrader, was able to reduce proliferation and induce apoptosis in EGFR-mutated NSCLC cell lines [135], suggesting that antibody-based PROTACs might potentially represent an alternative tool for fighting osimertinib resistance.

Even though PROTACs are showing promising preclinical results, clinical translation of these findings could be a challenge. Bioavailability and off-target actions of this type of molecule in patients must be considered. Currently, an EGFR PROTAC, namely HSK40118, is under evaluation in a phase 1 clinical trial in patients with EGFR-mutated NSCLC (NCT06050980). The results from this study might give important insights into the use of this relatively new class of inhibitors.

## 5. Strategies to Overcome Off-Target Resistance to Osimertinib (See Table 2)

### 5.1. MET

The most common alterations affecting MET in NSCLC can be summarized as exon 14 skipping mutations, protein overexpression, and gene amplification. As discussed earlier, amplification of MET has been reported as one of the most common mechanisms of resistance to EGFR TKIs, including osimertinib [80,81,136]. Hence, co-targeting MET and EGFR represents a good strategy to fight resistance to EGFR tyrosine kinase inhibitors [137]. Combinations of osimertinib and MET tyrosine kinase inhibitors have been extensively tested in case reports [138] and clinical trials. The combination of savolitinib, a highly selective MET TKI, and osimertinib has been tested in several clinical trials (TATTON, SAVANNAH, ORCHARD) in patients with amplification/overexpression of MET after failure of previous EGFR TKI treatment, showing promising results [139,140,141]. Of note, preliminary data from the SAVANNAH trial showed that the combination of osimertinib plus savolitinib is more effective in patients showing overexpression and/or amplification of MET, compared to the overall population [141], thus suggesting a possible role for MET levels as a biomarker predicting therapy response. The same combination is currently under evaluation in a phase 3 trial in the same subset of patients, compared to chemotherapy (SAFFRON, NCT05261399). Tepotinib is another MET TKI that is currently being investigated, in combination with osimertinib, in patients with EGFR-mutated lung cancer who acquired MET amplification after progressing to osimertinib. The INSIGHT 2 trial showed preliminary efficacy with an ORR of 50%, associated with an acceptable safety profile [142]. While MET amplification is one of the most common mechanisms of resistance to osimertinib in EGFR-mutated NSCLC, MET exon 14 skipping mutation has been rarely observed in this scenario. However, few case reports have shown the benefit of combining osimertinib with a MET TKI (tepotinib or crizotinib), following resistance to the third-generation TKI due to MET exon 14 skipping [143,144]. A retrospective analysis evaluated the outcome of capmatinib, yet another MET TKI, combined with osimertinib, in EGFR-mutated patients who developed MET alterations after first-line osimertinib. The study showed a clinical benefit associated with a manageable safety profile. In this study, of 17 patients who received the combination as second-line therapy and 1 patient in third-line therapy, 6% achieved complete response, 44% partial response, and 25% stable disease [145].

An alternative way to inhibit MET is represented by antibodies. In this respect, the development of amivantamab, a fully human bispecific antibody (bsAb) targeting MET and EGFR, represents a breakthrough in the managing of EGFR-mutated NSCLC. Amivantamab was first approved by the FDA in 2021 for patients with lung cancer carrying exon 20 insertion mutations in EGFR after the failure of chemotherapy [146]. The advantage of using antibodies instead of tyrosine kinase inhibitors could be attributed to their different mechanisms of action. Tyrosine kinase inhibitors bind to and block the kinase domain of the receptor, achieving a strong inhibition of its activation but also applying intense stress on the cell which might push towards the acquisition of mutations, potentially causing drug resistance. Antibodies, on the other hand, act via a completely different mechanism. Specifically, amivantamab inhibits ligand-induced receptor activation and promotes the downregulation of both EGFR and MET [147]. Additionally, this bispecific antibody stimulates Fc-dependent killing mechanisms mediated by immune cells, like antibody-dependent cellular cytotoxicity (ADCC) [147,148]. In the context of NSCLC expressing common EGFR mutations, amivantamab was tested in both first- and second-line settings. The MARIPOSA trial compared the combination of amivantamab plus lazertinib, a third-generation EGFR TKI, to osimertinib in untreated patients with lung cancer expressing either Del19- or L858R-EGFR. The combination treatment showed superior median PFS compared to osimertinib (23.7 vs. 16.6 months) [56]. Similarly, the MARIPOSA-2 trial showed an improved PFS for patients who progressed to osimertinib and received amivantamab plus chemotherapy with or without lazertinib compared to chemotherapy alone [57]. Based on the results of these two phase 2 clinical trials, the FDA granted approval to amivantamab for the treatment of patients with NSCLC expressing one of the two EGFR common mutations, in first-line settings and following resistance to osimertinib. The CHRYSALIS-2 trial further proved the efficacy of combining amivantamab and lazertinib in patients who progressed after osimertinib and platinum-based chemotherapy [55]. Data from this clinical trial showed that the combination was more beneficial for MET+ patients, identified by immunohistochemistry (IHC) staining, compared to MET- patients (ORR, 61% for MET+ vs. 12% for MET-) [149], emphasizing the importance of selecting subgroups of patients that may gain greater benefits from the combination therapy.

The antibody–drug conjugate (ADC) telisotuzumab vedotin (Teliso-V; ABBV-399) was developed from the humanized anti-MET antibody ABT-700 conjugated to the cytotoxic microtubule inhibitor monomethyl auristatin E (MMAE) and showed preclinical antitumor activity in MET-overexpressing or -amplified cell lines and patient-derived xenograft models [150]. When tested in a phase 1 study in 38 EGFR-mutated lung cancer patients who progressed on osimertinib treatment, the combination of telisotuzumab vedotin and osimertinib showed a median PFS of 7.4 months and an ORR of 50%, associated with a manageable safety profile [151], thus representing a potential additional tool for overcoming MET-mediated osimertinib resistance in EGFR-mutated NSCLC.

### 5.2. HER2

HER2 amplification is another common mechanism of resistance following treatment with an EGFR TKI, including first- and second-line osimertinib [80,81,152]. HER2 gene amplification leads to altered activation of the MAPK and PI3K pathways [153]. Unfortunately, there is no currently approved treatment available for HER2-mediated resistance to osimertinib in EGFR-mutated NSCLC. Preclinical evidence suggests that combining osimertinib with the anti-EGFR antibody cetuximab and the anti-HER2 antibody trastuzumab overcomes and prevents resistance to first- or second-line osimertinib in cell-line- and patient-derived xenograft models of EGFR-mutated NSCLC [154,155]. Similarly, the combination of osimertinib and the HER2 ADC trastuzumab emtansine (TDM1) was able to prevent or delay osimertinib resistance in preclinical models of EGFR-mutated lung cancer [156]. This combination has been evaluated in the phase 1/2 clinical trial TRAEMOS in patients with EGFR-mutated lung cancer that progressed on an EGFR TKI and showed HER2 amplification. Among 27 patients enrolled in the study, the ORR following 12 weeks of treatment was 4%, and the median PFS was 2.8 months [157]. Despite a manageable safety profile compared to cytotoxic chemotherapy, the efficacy of the combination was low, and the study was terminated. Other HER2 alterations reported for EGFR-mutated patients who progressed following osimertinib treatment include exon 20 insertion and exon 16 skipping mutations [95,158]. The ADC trastuzumab deruxtecan is the only HER2-targeting agent that has been approved by the FDA for the treatment of patients with HER2-mutated NSCLC who have previously received other systemic therapies [159]. In this regard, a recent case report shows that a patient with EGFR-mutated lung cancer who developed resistance to osimertinib and expressed exon 20 insertion mutation in HER2 gained approximately 8 months of benefit from the combination of the third-generation EGFR TKI and trastuzumab deruxtecan [160]. More studies are needed to elucidate if this HER2-targeting ADC is beneficial in the context of EGFR-mutated lung cancer.

### 5.3. HER3

Although genetic alterations involving HER3 are not described as canonical mechanisms of resistance to osimertinib, this receptor appears highly overexpressed in various malignant solid tumors, including lung cancer [161,162]. HER3 expression has been detected in 67% of circulating tumor cells from NSCLC patients, correlating with metastatic progression and decreased relapse-free survival [163]. Furthermore, elevated HER3 levels have been observed in EGFR-mutated lung cancer models following osimertinib treatment [164,165,166].

Previously, it has been demonstrated that the inhibition of one member of the ErbB family strongly results in the compensatory upregulation of the others prompting feedback loop activation through heterodimerization [154,167]. Indeed, due to its defective tyrosine kinase activity, HER3 is forced to heterodimerize. This feature makes this receptor especially competent to launch such bypass loops and dimerize with a variety of other RTKs such as HER2, MET, AXL, FGFR, and IGF1R [136,168,169,170]. HER3-containing heterodimers, especially with HER2, generate powerful survival signals and strongly stimulate the PI3K-AKT pathway, resulting in therapy resistance and bypassing EGFR inhibition. In line with these observations, neuregulin-1 (NRG1), the primary ligand for HER3, emerges as the strongest mitogenic factor in NSCLC [171] inducing HER2/HER3 coupling and signaling, thus supporting cancer cell survival, independently from the EGFR pathway [172].

In this context, the receptor has become a promising target for overcoming TKI resistance. Monoclonal antibodies (mAbs) targeting HER3, such as patritumab, seribantumab, elgemtumab, and lumretuzumab have been explored in both preclinical and clinical settings. Patritumab is a fully human mAb directed against the extracellular domain of HER3, competing with NRG1 for HER3 binding, thereby hindering the proliferation and survival of tumor cells [173,174]. Currently, the HER3-targeting antibody–drug conjugate (ADC) patritumab deruxtecan (HER3-DXd) has demonstrated the most significant anticancer activity against several tumors, including EGFR-mutated NSCLC [175,176,177]. In this approach, the HER3-targeting ADC is covalently linked to a topoisomerase I inhibitor, combining the selectivity of mAbs with the cytotoxic effect of a drug (payload) which is attached through a cleavable linker. A phase 1 dose-escalation/expansion study (NCT03260491) confirmed the safety and efficacy of HER3-DXd in patients with EGFR-mutated NSCLC with prior EGFR TKI therapy [178]. Subsequently, the phase 2 HERTENALung01 trial (NCT04619004) demonstrated an objective response rate of 29.8% and a median progression-free survival of 5.5 months in patients who had progressed on EGFR TKIs and platinum-based chemotherapy, regardless of the underlying resistance mechanisms [179]. Notably, HER3-DXd achieved a 33.3% ORR in non-irradiated brain metastases, addressing a critical need [179]. These encouraging data supported the launch of the phase 3 HERTENALung02 trial (NCT05338970) which aims to compare the safety and efficacy of HER3-DXd versus chemotherapy in patients with advanced EGFR-mutated NSCLC who have failed third-generation EGFR TKIs but have not yet received chemotherapy [180]. In addition, the phase 1 U31402-A-U103 (NCT04676477) dose-escalation and -expansion study is evaluating HER3-DXd in combination with osimertinib in first- and second-line settings for EGFR-mutated NSCLC, exploring innovative combinatory strategies to circumvent resistance [181].

Beyond monoclonal antibodies, bispecific antibodies targeting HER3 alongside other RTKs are under investigation. Zenocutuzumab, a bsAb targeting HER3 and HER2, has shown promising results, especially in disrupting HER2/HER3/NRG1 complex formation, and was recently approved by the FDA for NRG1-fusion-positive NSCLC [182]. The humanized bsAb targeting EGFR and HER3 duligotuzumab has demonstrated tumor growth inhibition in preclinical models of NSCLC and HNSCC (head and neck squamous cell carcinoma) resistant to erlotinib and cetuximab [183]. However, when tested in several phase 1/2 clinical trials, the bsAb showed limited activity [184,185,186,187].

Emerging therapeutic strategies also include HER3-targeted vaccines. For instance, a phase I clinical trial (NCT03832855) is evaluating pING-hHER3FL, a DNA vaccine encoding the full-length human HER3 protein, in patients with advanced or metastatic solid tumors. Lastly, targeting the HER3 ligand NRG1 could represent another promising approach. For example, 7E3 and YW538.24.71 are antibodies in the preclinical stage directed to the NRG1 IgG-like domain that blocks NRG1-dependent growth in pancreatic cancer models [188,189].

### 5.4. AXL

AXL is a receptor tyrosine kinase ubiquitously expressed in human tissues with a role in cell proliferation, migration, and adhesion [190]. Aberrations in this receptor have been reported in several types of cancer, including lung cancer, and have been associated with poor prognosis and resistance to cancer therapies [191]. It has been reported that patients with NSCLC with high levels of AXL mRNA exhibited shorter disease-free survival time compared to patients with low levels of AXL mRNA [192]. In the context of EGFR-mutated NSCLC, a study that analyzed 109 patients with lung adenocarcinoma showed that AXL expression correlated with lymph node metastasis and was more frequently detected in EGFR-mutated compared to EGFR wild-type lung tumors [193]. Several preclinical studies have reported that AXL mediates resistance to all generations of EGFR TKIs, including osimertinib, by sustaining the activation of survival pathways, such as the MAPK and AKT pathways, and that genetic or chemical inhibition of the receptor can overcome drug resistance [194,195,196,197]. Another mechanism that has been associated with resistance to EGFR TKIs is represented by the epithelial–mesenchymal transition (EMT) [198]. It has been shown that the downregulation of AXL in lung cancer cells resistant to EGFR TKIs reverts the EMT process and sensitizes the cells to tyrosine kinase inhibitors [199,200]. Moreover, AXL seems to have a role in the establishment of drug-tolerant persisters (DTPs), upon treatment with EGFR TKIs [170,201]. DTPs are cancer cells that survive following treatment with anticancer drugs, thanks to epigenetic mechanisms, and that can gradually accumulate genetic alterations that might lead to drug resistance [202,203,204]. Thus, blocking the generation of the persister cell population might be a strategy to prevent resistance to EGFR TKIs. Of note, increased expression of AXL and its ligand GAS6 has been detected in samples from patients with EGFR-mutated lung cancer who acquired resistance to EGFR TKIs [194]. Moreover, increased levels of AXL mRNA have been detected in circulating tumor cells (CTCs) obtained from patients after one cycle of osimertinib and at disease progression compared to baseline [205]. All this evidence suggests a potential benefit of combining EGFR and AXL inhibitors to fight EGFR TKI resistance. In this regard, several preclinical studies have shown that resistance to EGFR TKIs could be prevented by co-administering AXL-targeting agents, including TKIs, monoclonal antibodies, and bispecific antibodies [197,206,207,208]. In line with these findings, several AXL inhibitors have entered clinical evaluation in the setting of EGFR-mutated NSCLC resistant to EGFR TKIs [209].

### 5.5. FGFR

The fibroblast growth factor receptor (FGFR) family comprises five transmembrane RTKs (FGFR1–5). Among them, FGFR1-4 share a highly conserved structure and can conduct signal transmission after binding with ligands. In contrast, FGFR5 lacks the intracellular catalytic domain, and it is unable to conduct signal transduction. The activation of FGFRs through fibroblast growth factor (FGF) ligands triggers a cascade of intracellular pathways, mainly the PI3K/AKT and MAPK signaling routes [210]. Their activation culminates in different cell fate decisions such as cell survival, motility and invasiveness, cell proliferation, EMT, and angiogenesis [210]. In several cancer types, FGFR signaling becomes dysregulated through both ligand-dependent and -independent mechanisms [211,212,213]. The most common alterations include FGFR amplification, mutations, and translocations, leading to FGFR overexpression and constitutive tyrosine kinase activation [214].

In NSCLC, FGFR has rapidly gained attention as a mechanism of resistance to osimertinib. Indeed, several studies have highlighted its critical role in the formation of DTPs following EGFR TKI therapy and its involvement in promoting EMT as a survival strategy [215,216]. The most significant reported alterations are FGFR1 amplification and FGFR3 fusions, which suggest that abnormal FGFR signaling could be a potential therapeutic target for overcoming osimertinib resistance [217]. For instance, dual targeting of FGFR and AKT with respective inhibitors has shown promise in FGFR1-overexpressing cells [218]. In addition, a combinatory therapy targeting multiple pathways, including osimertinib plus an AXL inhibitor (ONO-7475) and an FGFR inhibitor (BGJ398), has demonstrated marked antitumor effects by reducing cell viability compared to dual therapy. In xenograft models, this triple inhibition strongly suppressed tumor re-growth, suggesting that an initial blockade of FGFR1 may be pivotal for preventing resistance [219]. Furthermore, dual targeting of EGFR and FGFR pathways has proven effective in overcoming acquired resistance to EGFR TKIs in NSCLC [216,220]. Several FGFR inhibitors, including AZD4547, nintedanib, anlotinib, erdafitinib, and pemigatinib, are currently under investigation in clinical trials for their potential in treating NSCLC patients harboring FGFR alterations [221,222]. Notably, case reports have demonstrated that erdafitinib can overcome FGFR3-TACC3-mediated resistance to osimertinib. Indeed, two osimertinib-resistant patients treated with erdafitinib experienced clinical benefits lasting 13.0 and 6.0 months, respectively, demonstrating the potential of FGFR inhibition in reversing resistance [223,224]. Therefore, the addition of erdafitinib in the treatment of patients with FGFR abnormalities who are progressing on osimertinib might be crucial, and prospective clinical trials are warranted to validate this approach.

### 5.6. VEGF/VEGFR

Among different strategies proposed to bypass TKI resistance in EGFR-mutated NSCLC, targeting the VEGFR (vascular endothelial growth factor receptor) pathway appears to be an interesting approach. VEGFRs belong to the type V RTK family, which includes three variants, with VEGFR1 and, more notably, VEGFR2 being the most significant expressed on vascular endothelial cells [225]. Pathway activation occurs upon the binding of VEGF ligands (VEGF-A, B, C, and D), a group of polypeptide growth factors belonging to the VEGF-PDGF (platelet-derived growth factor) supergene family. In particular, VEGF-A, following binding to VEGFR2, mediates pro-angiogenic signals, sustaining cancer cell growth by promoting the formation of new blood vessels [226]. In light of this, several drugs, including monoclonal antibodies and TKIs, targeting the VEGF-VEGFR axis have been developed. In particular, bevacizumab, a humanized anti-VEGF-A antibody, and ramucirumab, a fully human mAb targeting VEGFR2, are currently approved in advanced NSCLC [227,228].

VEGF and EGFR signaling share several downstream pathways. In addition, in models of EGFR-mutated NSCLC, EGFR activation leads to increased expression of VEGF, which in turn can contribute to the reduction in EGFR TKI efficacy [228]. In this scenario, clinical trials, combining erlotinib with an anti-angiogenic agent in first-line settings, have shown improved PFS for EGFR-mutated NSCLC patients [229,230]. Of note, the combination of erlotinib with either bevacizumab or ramucirumab is approved as first-line therapy for the treatment of patients with NSCLC harboring canonical EGFR mutations (Del19 or L858R) in Europe and the USA, respectively. This suggests that targeting the VEGFR pathway could also be beneficial in preventing resistance to osimertinib. In this regard, a phase 1/2 clinical trial testing the combination of osimertinib and bevacizumab in 49 patients with EGFR-mutated lung cancer showed promising results, with a PFS at 12 months of 76% and an ORR of 80% [231]. However, the largest studies have failed to demonstrate an improvement in terms of PFS and OS when comparing the combination of the two agents to osimertinib monotherapy in both first- and second-line therapy [232,233,234]. A phase 2 trial is currently investigating the safety and efficacy of osimertinib plus bevacizumab in treatment-naïve patients with NSCLC expressing L858R-EGFR [235]. The results from this study might give insights into the advantages of selecting subgroups that might benefit from this combination therapy. More promising results arose from the combination of osimertinib with the anti-VEGFR2 mAb ramucirumab. The RAMOSE phase 2 trial, enrolling TKI-naïve EGFR-mutated NSCLC patients, showed a median PFS of 24.8 months for the combination therapy versus 15.6 months in the osimertinib-treated group, with a manageable safety profile for the combination [236].

### 5.7. IGF1R

For all three generations of EGFR TKIs, bypass activation of the insulin-like growth factor 1 receptor (IGF1R), which sustains the activation of the AKT and MAPK pathways, has been described as a mechanism of drug resistance in preclinical models of EGFR-mutated NSCLC [237,238,239,240,241,242,243]. Importantly, evidence from the literature indicates that IGF1R plays a major role in the generation of DTPs following treatment with EGFR TKIs and that inhibition of the receptor abrogates the establishment of the drug-tolerant cell population, thus, potentially blocking the onset of resistance [203,244]. Of clinical interest, elevated levels of activated IGF1R have been detected in tumor samples obtained from patients with EGFR-mutated NSCLC who developed resistance to osimertinib [239,240]. In this respect, several preclinical studies have shown that simultaneously inhibiting EGFR and IGF1R, via genetic inhibition or by using small-molecule inhibitors, represents a valid strategy to overcome resistance to EGFR TKIs [240,243,245]. Unfortunately, when the IGF1R inhibitor linsitinib was combined with erlotinib in EGFR-mutated lung cancer patients, the PFS, ORR, and disease control rate were lower in the combination group compared to the erlotinib monotherapy group [246]. More studies are needed to investigate the potential role of biomarkers that could predict response to IGF1R inhibitors in the setting of EGFR-mutated NSCLC.

### 5.8. Other Strategies

Oncogenic fusions involving ALK and RET have been identified in patients who progressed after first- or second-line osimertinib [80,81,247]. Several case reports have shown the benefit of combining osimertinib with inhibitors of ALK and RET, when oncogenic fusions were detected as a mechanism of resistance to osimertinib [248,249,250]. Additionally, patients with EGFR-mutated lung cancer, positive for RET fusions at the time of progression on osimertinib, who received the combination of the third-generation TKI and the RET inhibitor selpercatinib, gained clinical benefit associated with a safety profile [251]. Moreover, the ORCHARD phase II clinical was designed to test several osimertinib-based combinatorial approaches following resistance to first-line osimertinib [115]. The combinations include, as mentioned above, osimertinib plus savolitinib, when MET amplification was detected; osimertinib and alectinib, when ALK rearrangement was detected, and osimertinib and selpercatinib in the presence of RET rearrangement.

Alterations in EGFR downstream signaling molecules have also been reported in patients who acquired resistance to osimertinib [80,81]. In this respect, co-targeting EGFR and the altered molecules might represent a valid strategy to prevent drug resistance. Notably, when BRAF V600E mutation occurs following osimertinib treatment, an analysis of case reports showed the efficacy (a median PFS of 7 months following osimertinib progression, an OS of 46.2 months, and initial response of 60%) and acceptable tolerability of the triple combination including osimertinib, the BRAF inhibitor dabrafenib, and the MEK inhibitor trametinib [252].

Although immune checkpoint inhibitors (ICIs) have revolutionized cancer treatment, advantages for advanced NSCLC, in terms of OS, have been reported for EGFR wild-type tumors, but not for EGFR-mutated lung tumors [253]. The role of ICIs in overcoming acquired drug resistance in the settings of EGFR-mutated NSCLC has also been investigated. While ICI single agents do not show any benefit in resistance settings, their combination with chemotherapy and anti-angiogenic agents might be beneficial [254]. However, an increased risk of severe adverse effects has been reported for patients receiving osimertinib and ICIs, leading to clinical trial termination [255,256]. Given the controversial results in terms of efficacy and the increased risk of toxicity with ICI–osimertinib-based regimens, immunotherapy is unlikely to provide significant benefit in EGFR-mutated NSCLC at present.

## 6. Conclusions

The third-generation EGFR TKI osimertinib has greatly improved the management of patients with lung cancer driven by EGFR activating mutations, both as first- and second-line therapy. Unfortunately, drug resistance, due to on- or off-target mechanisms, occurs. When specific mechanisms of resistance are identified following tissue or liquid biopsy, further targeted therapies can be employed. However, managing patients with unknown mechanisms of resistance remains more challenging. For therapy failure due to the appearance of the mutation C797S in the kinase domain of EGFR, several strategies aimed at preventing or overcoming resistance are under development. These include the development of fourth-generation EGFR TKIs and EGFR-targeting PROTACs. Even though several of these new inhibitors are under clinical evaluation, none of them has been granted clinical approval. Another aspect to consider regarding new-generation EGFR TKIs is the potential resistance that could arise following treatment with these small molecule inhibitors, similar to what has been observed with all previous-generation TKIs. When considering off-target mechanisms of resistance, thus far, the most successful strategy has been the dual targeting of EGFR and MET. In this regard, the MARIPOSA and MARIPOSA-2 trials represent a milestone in the field of EGFR-mutated NSCLC, leading to the approval of the combination of a third-generation EGFR TKI and the bispecific EGFR-MET antibody amivantamab, in both first- and second-line settings. The approval of amivantamab paves the way for more studies involving other bispecific antibodies, for instance targeting EGFR and AXL, or other receptors involved in resistance to osimertinib. On one hand, it is becoming increasingly clear that combination therapies represent the path to follow to fight drug resistance. On the other hand, another important factor to consider is the selection of patients who would benefit from a specific therapy. For instance, L858R-EGFR lung cancer models seem to be more sensitive to therapies involving anti-EGFR monoclonal antibodies compared to Del19-EGFR-expressing models [72]. In this scenario, the selection of the proper therapy would greatly benefit from the identification of biomarkers that predict treatment response and real-time monitoring of the patient’s response to the treatment.

## Figures and Tables

**Table 1:** Drugs approved by the FDA for the treatment of EGFR-mutated NSCLC.

Name of the Molecule	Trade Name	Class	Year of FDA Approval	Company	Indication	Key Clinical Studies
Gefitinib	Iressa	1st generation TKI	2003	AstraZeneca	Del19/L858R-EGFR positive NSCLC	NEJ002 [34,35] IPASS [36,37] WJTOG3405 [38,39]
Erlotinib	Tarceva	1st generation TKI	2004	OSI Pharms	Del19/L858R-EGFR positive NSCLC	OPTIMAL [40,41] EUTARC [42]
Afatinib	Gilotrif	2nd generation TKI	2013	Boehringer Ingelheim	Del19/L858R-EGFR positive NSCLC	LUX-Lung3 [43,44] LUX-Lung6 [43,45]
Osimertinib	Tagrisso	3rd generation TKI	2015	AstraZeneca	Del19/L858R/T790M-EGFR positive NSCLC	AURA3 [46,47] FLAURA [48,49]
Dacomitinib	Vizimpro	2nd generation TKI	2018	Pfizer	Del19/L858R-EGFR positive NSCLC	ARCHER1050 [50,51,52]
Amivantamab	Rybrevant	Bispecific Antibody	2021	Janssen Biotech	Ins20/Del19/L858R-EGFR positive NSCLC	PAPILLON [53] CHRYSALIS [54] CHRYSALIS2 [55] MARIPOSA [56] MARIPOSA2 [57]
Mobocertinib	Exkivity	TKI	2021 (withdrawn in 2024)	Takeda Pharms	Ins20-EGFR positive NSCLC	NCT02716116 [58]
Lazertinib	Lazcluze	3rd generation TKI	2024	Janssen Biotech	Del19/L858R-EGFR positive NSCLC	MARIPOSA [56] MARIPOSA2 [57]

Note: The abbreviations used are as follows: FDA, Food and Drug Administration; TKI, tyrosine kinase inhibitor; Del19, deletion mutations in exon 19; NSCLC, non-small-cell lung cancer; Ins20, insertion mutations in exon 20.

**Table 2:** Clinical strategies addressing osimertinib resistance in EGFR-mutated NSCLC.

Mechanism of Resistance	Player of Resistance	Strategy to Overcome Resistance	Drugs	Status	Relevant Studies
ON-TARGET	EGFR	EGFR TKI (4th generation)	JIN-A02	Phase 1–2 (recruiting)	NCT05394831
			HS-10375	Phase 1–2 (recruiting)	NCT05435248
			WJ13405	Phase 1–2 (recruiting)	NCT05662670
			BLU-945	Phase 1–2 (active, not recruiting)	NCT04862780 (SYMPHONY)
			BPI-361175	Phase 1–2 (not yet recruiting)	NCT05393466
			BDTX-1535	Phase 1–2 (recruiting)	NCT05256290
			QLH11811	Phase 1 (recruiting)	NCT05555212
			H002	Phase 1–2 (recruiting)	NCT05552781
		EGFR/HER2 TKI	BAY2927088	Phase 1–2 (active, not recruiting)	NCT05099172
		1st- + 3rd-generation EGFR TKI	Osimertinib + gefitinib	Phase 1–2 (active, not recruiting)	NCT03122717
		1st- + 3rd-generation EGFR TKI	Osimertinib + gefitinib	Phase 2 (active, not recruiting)	NCT03944772 (ORCHARD)
		2nd- + 3rd-generation EGFR TKI	Osimertinib + dacomitinib	Early phase 1 (completed)	NCT03755102
		3rd-generation EGFR TKI + EGFR mAb	Osimertinib + necitumumab	Phase 2 (active, not recruiting)	NCT03944772 (ORCHARD)
		EGFR PROTAC	HSK40118	Phase 1 (recruiting)	NCT06050980
OFF-TARGET	MET	MET TKI	Osimertinib + savolitinib	Phase 1 (active, not recruiting)	NCT02143466 (TATTON)
			Osimertinib + savolitinib	Phase 2 (active, not recruiting)	NCT03778229 (SAVANNAH)
			Osimertinib + savolitinib	Phase 3 (recruiting)	NCT05015608 (SACHI)
			Osimertinib + savolitinib	Phase 2 (active, not recruiting)	NCT03944772 (ORCHARD)
			Osimertinib + savolitinib	Phase 2 (active, not recruiting)	NCT05163249 (FLOWERS)
			Osimertinib + savolitinib	Phase 3 (recruiting)	NCT05261399 (SAFFRON)
			Osimertinib + savolitinib	Phase 2 (active, not recruiting)	NCT04606771
			Osimertinib + savolitinib	Phase 3 (recruiting)	NCT05009836 (SANOVO)
			Osimertinib + tepotinib	Phase 2 (active, not recruiting)	NCT03940703 (INSIGHT 2)
			Osimertinib + vebreltinib	Phase 1–2 (recruiting)	NCT04743505
			Osimertinib + glumetinib	Phase 1–2 (unknown status)	NCT04338243
			Osimertinib + capmatinib	Phase 3 (terminated)	NCT04816214 (GEOMETRY-E)
			Osimertinib + capmatinib ± ramucirumab	Phase 2 (recruiting)	NCT05642572
		Bispecific antibody (EGFR-MET)	Lazertinib + amivantamab + bevacizumab	Phase 2 (recruiting)	NCT05601973 (AMAZE-Lung)
			Osimertinib + EMB-01	Phase 1–2 (not yet recruiting)	NCT05498389
			MCLA-129 + Befotertinib (3rd-generation EGFR TKI)	Phase 1 (not yet recruiting)	NCT06015568
		MET ADC	Osimertinib + telisotuzumab vedotin	Phase 1 (active, not recruiting)	NCT02099058
	HER2	HER2 ADC	Osimertinib + trastuzumab-emtansine	Phase 2 (terminated)	NCT03784599 (TRAEMOS)
		HER2 mAb	Osimertinib + necitumumab + trastuzumab	Phase 1–2 (active, not recruiting)	NCT04285671
	HER3	HER3 ADC	Patritumab deruxtecan	Phase 1 (recruiting)	NCT03260491
			Patritumab deruxtecan	Phase 2 (active, not recruiting)	NCT04619004 (HERTHENA-Lung01)
			Patritumab deruxtecan	Phase 3 (active, not recruiting)	NCT05338970 (HERTHENA-Lung02)
			Osimertinib + patritumab deruxtecan	Phase 1 (active, not recruiting)	NCT04676477
		Bispecific antibody (EGFR-HER3)	Osimertinib + izalontamab	Phase 2–3 (recruiting)	NCT05020769
		EGFR-HER3 ADC (bispecific antibody)	Osimertinib + BL-B01D1	Phase 2 (recruiting)	NCT05880706
			Osimertinib + BMS-986507	Phase 1–2 (recruiting)	NCT06618287
	AXL	AXL TKI	Dubermatinib (TP-0903)	Phase 1 (completed)	NCT02729298
			Osimertinib + DS-1205c	Phase 1 (terminated)	NCT03255083
	Downstream molecules	BRAF + MEK inhibitor	Dabrafenib + trametinib	Phase 2 (completed)	NCT04452877
		mTOR inhibitor	Osimertinib + sapanisertib	Phase 1 (active, not recruiting)	NCT02503722
			Osimertinib + sapanisertib	Phase 1 (completed)	NCT04479306
		JAK inhibitor	Osimertinib + itacitinib	Phase 1–2 (active, not recruiting)	NCT02917993
			Osimertinib + golidocitinib	Phase 1–2 (completed)	NCT03450330 (JACKPOT1)
		MEK inhibitor	Osimertinib + selumetinib	Phase 1 (active, not recruiting)	NCT02143466
			Osimertinib + selumetinib	Phase 2 (active, not recruiting)	NCT03392246
			Osimertinib + selumetinib	Phase 2 (active, not recruiting)	NCT03944772 (ORCHARD)
		PI3K inhibitor	Osimertinib + TQ-B3525	Phase 1–2 (unknown status)	NCT05284994
	ALK	ALK TKI	Osimertinib + alectinib	Phase 2 (active, not recruiting)	NCT03944772 (ORCHARD)
	RET	RET TKI	Osimertinib + selpercatinib	Phase 2 (active, not recruiting)	NCT03944772 (ORCHARD)
	Cell cycle/apoptosis regulators	CDK 4/6 inhibitor	Osimertinib + G1738 (lerociclib)	Phase 1–2 (completed)	NCT03455829
			Osimertinib + abemaciclib	Phase 2 (active, not recruiting)	NCT04545710
		Bcl-2 inhibitor	Osimertinib + navitoclax	Phase 1 (completed)	NCT02520778
			Osimertinib + pelcitoclax	Phase 1 (recruiting)	NCT04001777
		Aurora Kinase A inhibitor	Osimertinib + VIC-1911	Phase 1 (recruiting)	NCT05489731
			Osimertinib + alisertib	Phase 1 (completed)	NCT04479306
			Osimertinib + alisertib	Phase 1 (recruiting)	NCT04085315
			Osimertinib + LY3295668	Phase 1–2 (active, not recruiting)	NCT05017025
	VEGF/VEGFR	VEGF mAb	Osimertinib + bevacizumab	Phase 2 (completed)	NCT03133546 (BOOSTER)
			Osimertinib + bevacizumab	Phase 1–2 (completed)	NCT02803203
			Osimertinib + bevacizumab	Phase 3 (recruiting)	NCT04181060
			Osimertinib + bevacizumab	Phase 3 (recruiting)	NCT05104281
			Osimertinib + bevacizumab	Phase 2 (active, not recruiting)	NCT02971501
			Osimertinib + bevacizumab	Phase 2 (not yet recruiting)	NCT04988607
		VEGFR mAb	Osimertinib + ramucirumab	Phase 2 (active, not recruiting)	NCT03909334 (RAMOSE)
			Osimertinib + ramucirumab or necitumumab	Phase 1 (completed)	NCT02789345
			Osimertinib + ramucirumab	Phase 3 (active, not recruiting)	NCT02411448 (RELAY)
	Others	VEGFR-PDGFR-FGFR-cKIT TKI	Osimertinib + Anlotinib	Phase 1–2 (completed)	NCT04770688 (AUTOMAN)
		MERTK and FLT3 TKI	Osimertinib + MRX-2843	Phase 1 (recruiting)	NCT04762199
		ROS1/TRK/ALK TKI	Osimertinib + repotrectinib	Phase 1 (recruiting)	NCT04772235 (TOTEM)

Note: The abbreviations used are as follows: TKI, tyrosine kinase inhibitor; mAb, monoclonal antibody; ADC, antibody–drug conjugate.
